# Identification of an Antiviral Compound from the Pandemic Response Box that Efficiently Inhibits SARS-CoV-2 Infection In Vitro

**DOI:** 10.3390/microorganisms8121872

**Published:** 2020-11-26

**Authors:** Melle Holwerda, Philip V’kovski, Manon Wider, Volker Thiel, Ronald Dijkman

**Affiliations:** 1 Institute of Virology and Immunology, 3147 Mittelhäusern, Switzerland; melle.holwerda@vetsuisse.unibe.ch (M.H.); Philip.vkovski@ifik.unibe.ch (P.V.); volker.thiel@vetsuisse.unibe.ch (V.T.); 2 Department of Infectious Diseases and Pathobiology, Vetsuisse Faculty, University of Bern, 3012 Bern, Switzerland; 3 Institute for Infectious Diseases, University of Bern, 3001 Bern, Switzerland; manon.wider@ifik.unibe.ch; 4 Graduate School for Molecular Cell Biology, University of Bern, 3012 Bern, Switzerland

**Keywords:** pandemic response box, drug repurposing, compound screen, SARS-CoV-2, Retro-2.1, remdesivir, Vero E6, Calu-3

## Abstract

With over 50 million currently confirmed cases worldwide, including more than 1.3 million deaths, the severe acute respiratory syndrome coronavirus 2 (SARS-CoV-2) pandemic has a major impact on the economy and health care system. Currently, limited prophylactic or therapeutic intervention options are available against SARS-CoV-2. In this study, 400 compounds from the antimicrobial “pandemic response box” library were screened for inhibiting properties against SARS-CoV-2. An initial screen on Vero E6 cells identified five compounds that inhibited SARS-CoV-2 replication. However, validation of the selected hits in a human lung cell line highlighted that only a single compound, namely Retro-2.1, efficiently inhibited SARS-CoV-2 replication. Additional analysis revealed that the antiviral activity of Retro-2.1 occurs at a post-entry stage of the viral replication cycle. Combined, these data demonstrate that stringent in vitro screening of preselected compounds in multiple cell lines refines the rapid identification of new potential antiviral candidate drugs targeting SARS-CoV-2.

## 1. Introduction

In December 2019, a new zoonotic coronavirus emerged in Wuhan, Hubei province, China, named severe acute respiratory syndrome coronavirus 2 (SARS-CoV-2), which is the etiological agent of coronavirus disease 2019 (COVID-19) [1,2,3]. The clinical features of SARS-CoV-2-infected patients range from mild cold-like symptoms to severe illness ultimately leading to acute respiratory distress syndrome [2,4]. Patients at an older age and with underlying comorbidities are at higher risk of developing severe courses of COVID-19 [5]. Despite unprecedented international public health response measures to contain SARS-CoV-2 transmissions, the viral outbreak is currently categorized as a pandemic with over 50 million confirmed laboratory cases reported worldwide, including over 1.3 million deaths as of November 2020 [6]. At present, and despite earlier outbreaks of SARS-CoV and Middle East respiratory syndrome (MERS)-CoV, there are limited approved antiviral treatment options, such as antiviral drugs, vaccines and immuno-prophylaxis, that can be used prophylactically or therapeutically to halt the current SARS-CoV-2 infections.

Vaccine development is a long process to reach approvement for clinical use, and although SARS-CoV-2 vaccine developments are currently expedited, the eventual worldwide vaccine distribution may be delayed for additional months [7]. Moreover, while vaccines are used prophylactically, antiviral drugs can be employed both prophylactically and therapeutically. For SARS-CoV-2, several antiviral compounds have been evaluated, such as the nucleoside analogue remdesivir, the transmembrane serine protease 2 (TMPRSS2)inhibitor camostat mesylate, and the antimalaria drug (hydroxy-) chloroquine, targeting different stages of the viral replication cycle [8,9,10]. All three antiviral drugs have recently been tested in large patient cohorts, whereby remdesivir has been shown to reduce the hospitalization time, but proved to exert only a marginal benefit for patients with severe COVID-19 disease [11]. Several clinical trials with either hydroxychloroquine or chloroquine did not reveal any benefit in the clinical outcome [11,12,13,14,15]. Additionally, RNA viruses, including coronaviruses, are known to rapidly evade antiviral drug inhibition by developing resistance mutations and subsequent selection of drug-resistant viral populations [16,17,18]. Therefore, the use of multiple drug regimens as well as expanding the repertoire of available antiviral treatment options are of crucial importance to combat the SARS-CoV-2 pandemic.

Since the beginning of the 21st century, multiple epidemics have been caused by a viral or bacterial agent, such as the Ebola-, measles-, Zika-viruses and cholera [19]. Some epidemics have even reached pandemic proportions, such as the influenza A/H1N1 virus and the currently circulating SARS-CoV-2 [20,21]. As a rapid response to these virulent agents, the Medicines for Malaria Venture and Drugs for Neglected Diseases initiatives developed the pandemic response box (PRB), a compound library containing 400 compounds with antibacterial, antifungal and antiviral properties. This compound library allows rapid evaluation of potential drug repurposing against newly emerging pathogens.

To this end, we performed an in vitro-based screen of 400 preselected compounds with antibacterial, antifungal, and antiviral properties contained in the PRB and assessed their antiviral activity against SARS-CoV-2. A stringent large-scale screen in Vero E6 cells highlighted sixteen compounds that prevented virus-induced cytopathogenic effects (CPE) while displaying low cytotoxicity and no detrimental effect on cell viability. Further validation using Vero E6 and Calu-3 cells revealed that only one compound, Retro-2.1, showed substantial SARS-CoV-2 inhibition while the antiviral effects of the other compounds were only observed in Vero E6 cells. Time-of-addition analysis indicated that Retro-2.1 targets SARS-CoV-2 at a post-entry stage of the viral replication cycle. Together, these data demonstrate that stringent in vitro screening of preselected compounds on different cell lines refines the rapid identification of a new potential antiviral candidate drugs targeting SARS-CoV-2.

## 2. Materials and Methods

### 2.1. Cell Lines and Viruses

Vero E6 cells (kindly provided by M. Müller and C. Drosten, Charité, Berlin, Germany) and Calu-3 cells (HTB-55, American Type Culture Collection (ATCC), Manassas, VA, USA) were propagated in Dulbecco’s modified Eagle Medium–GlutaMAX, 10% (*v/v*) heat-inactivated fetal bovine serum, 100 mg/mL streptomycin, 100 IU/mL penicillin, 1% (*w/v*) non-essential amino acids and 15 mM 4-(2-hydroxyethyl)-1-piperazineethanesulfonic acid (HEPES, Gibco, Gaithersburg, MD, USA). Cells were maintained at 37 °C in a humidified incubator with 5% CO_2_. SARS-CoV-2 (SARS-CoV-2/München-1.1/2020/929 [22]) stocks were produced on Vero E6 cells, aliquoted and stored at −80 °C. Viral titers were determined by tissue culture infectious dose 50 (TCID_50_) on Vero E6 cells after 72 h incubation at 37 °C in a humidified incubator with 5% CO_2_.

### 2.2. Compound Preparation of the Pandemic Response Box

All 400 compounds (purity of >90%) distributed in 96-well plates were dissolved and diluted in dimethyl sulfoxide (DMSO, Sigma-Aldrich, St. Louis, MO, USA) and aliquoted at a concentration of 1 mM in 96-well plates (Techno Plastic Products AG, Trasadingen, Switzerland) and were kept at −20 °C until further use. Compounds were diluted at the indicated concentration in cell culture medium. The control compound remdesivir (MedChem Express, NJ, USA, HY-104077) was diluted to a 20 mM working stock in DMSO and stored at −80 °C, while K22 was prepared and stored as described previously [17].

### 2.3. Antiviral Activity Screening

Vero E6 cells were seeded in 96-well clear bottom, black wall plates (Costar, Tewksbury, MA, USA), at a density of 20,000 cells per well, one day prior to the experiment. Cells were pretreated for 2 h with 1 µM of each compound contained in the PRB. Remdesivir [8], K22 [17], and vehicle controls (DMSO) were included in each plate. Subsequently, cells were infected with SARS-CoV-2 at a multiplicity of infection (MOI) of 0.01 in compound-containing medium and incubated at 37 °C in a humidified incubator with 5% CO_2_. Uninfected (mock) controls were included in each plate. At 48 h post-infection (hpi), cells were fixed with 4% (*v/v*) neutral buffered formalin (Formafix AG, Hittnau, Switzerland) and stained with crystal violet. Cell viability and cytotoxicity were assessed in parallel, in identically treated, uninfected plates. Two independent experiments were performed, each including a technical duplicate. Wells containing an intact cell layer without apparent CPE after infection and displaying high cell viability and low cytotoxicity were considered as hits.

### 2.4. Cell Cytotoxicity and Cell Viability

Cell cytotoxicity and viability were assessed using CellTox™ green cytotoxicity assay (Promega, Dübendorf, Switzerland) and CellTiter-Glo® 2.0 assay (Promega), respectively, according to manufacturer’s protocols. Readout was performed on a Cytation 5 Cell Imaging Multi-Mode Reader (BioTek, Sursee, Switzerland). The mean raw values were standardized by calculating the z-scores and individual cell cytotoxicity and viability scores of compounds exceeding a confidence interval of 95% (z-score of −2 or +2) were regarded as nonviable hits.

### 2.5. Half Maximal Effective Concentration (EC_50_) Determination of Selected Compounds

Vero E6 and Calu-3 cells were seeded in 96-well clear bottom, black wall plates (Costar), at a density of 20,000 or 40,000 cells per well, respectively, one day prior to the experiment. Cells were pretreated for 2 h with 2-fold serial dilutions of selected compounds, ranging from 4 µM to 0.062 µM. Cells were infected with SARS-CoV-2 (MOI of 0.01) in compound-containing medium and incubated at 37 °C in a humidified incubator with 5% CO_2_. Cells were fixed with 4% (*v/v*) neutral buffered formalin at 24 and 48 (Vero E6) or 48 (Calu-3) hours post-infection and processed for immunofluorescence analysis. Briefly, cells were permeabilized with 0.1% (*v/v*) Triton X-100 (Merck, Kenilworth, NJ, USA) for 5 min and blocked in phosphate-buffered saline (PBS), supplemented with 50 mM NH_4_Cl, 0.1% (*w/v*) saponin (Sigma) and 2% (*w/v*) bovine serum albumin (Immunoglobulin G (IgG)-free, Jackson Immunoresearch, Westgrove, PA, USA). SARS-CoV-2 antigen-positive cells were detected using a rabbit polyclonal anti-SARS-CoV nucleocapsid protein (Rockland, Limerick, PA, USA 200-401-A50) and a secondary Alexa Fluor^®^ 488-labeled donkey anti-rabbit IgG (H + L) or Alexa Fluor^®^ 647-labeled donkey anti-rabbit IgG (H + L) (Jackson Immunoresearch). Samples were counterstained using 4′,6-diamidino-2-phenylindole (DAPI, Thermo Fisher Scientific, Darmstadt, Germany) to visualize the nuclei and finally washed with PBS.

Images were acquired on a Cytation 5 Cell Imaging Multi-Mode Reader (BioTek) equipped with a 4× air objective (numerical aperture: 0.13). Four images per well were acquired to cover the entire surface of the well and processed and stitched using the Gen5 ImagePrime software package (v. 3.08.01). The percentage of virus-infected cells in each well was calculated by dividing the number of cells with a cytoplasmic green fluorescent protein (GFP) signal (SARS-CoV nucleocapsid) by the total number of detected cells (DAPI). Nuclei were segmented using a primary mask with dynamic thresholding and a secondary mask encompassing the cytoplasm was based on the primary mask, dilated by 5 µm. The half maximal effective concentration (EC_50_) was calculated in GraphPad Prism (San Diego, CA, USA, v. 9.0.0) using the non-linear variable slope with four parameters option, the corresponding EC_90_-value was calculated based on the previously calculated EC_50_-value and the hill slope values.

### 2.6. Half-Maximum Cytotoxicity Concentration (CC_50_) Determination

To determine the cytotoxicity of the compounds and for later calculation of the selectivity index (SI), a dilution series ranging from 100 μM to 0.19 μM for both Vero E6 cells and Calu-3 cells was prepared in cell culture medium and incubated for 48 h at 37 °C in a humidified incubator with 5% CO_2_. Cytotoxicity and cell viability were both assessed as described earlier, whereby the CC_50_ was calculated in GraphPad Prism (v. 9.0.0) using the non-linear variable slope with four parameters option.

### 2.7. Time-of-Addition Experiment

Vero E6 cells were seeded in 96-well, clear bottom, black wall plates (Costar) at a density of 20,000 cells per well. Retro-2.1 and the vehicle control (DMSO) were diluted in cell culture medium at a concentration of 10 μM or using an equal volume of DMSO. Cells were (pre-) treated and infected with a MOI of 0.01. Following each incubation step, cells were washed three times with PBS to remove any residual compound or virus. At 24 h post-infection, supernatant was collected for viral titration, while cells were fixed and processed for immunofluorescence analysis as described before using an Alexa Fluor^®^ 647-labeled donkey anti-rabbit IgG (H + L) as a secondary antibody (Jackson Immunoresearch).

### 2.8. Data Representation

Graphs were generated using GraphPad Prism software (v. 9.0.0) and the final figures were assembled in Adobe Illustrator CS6 (v. 16.0.0). Brightness and contrast of microscopy pictures were minimally adjusted and processed identically to their corresponding control using FIJI (v. 1.53c). Images were assembled using the FigureJ plugin in FIJI [23].

## 3. Results

### 3.1. Survival Screen with Compounds Included in the Pandemic Response Box against SARS-CoV-2

**Figure 1:**
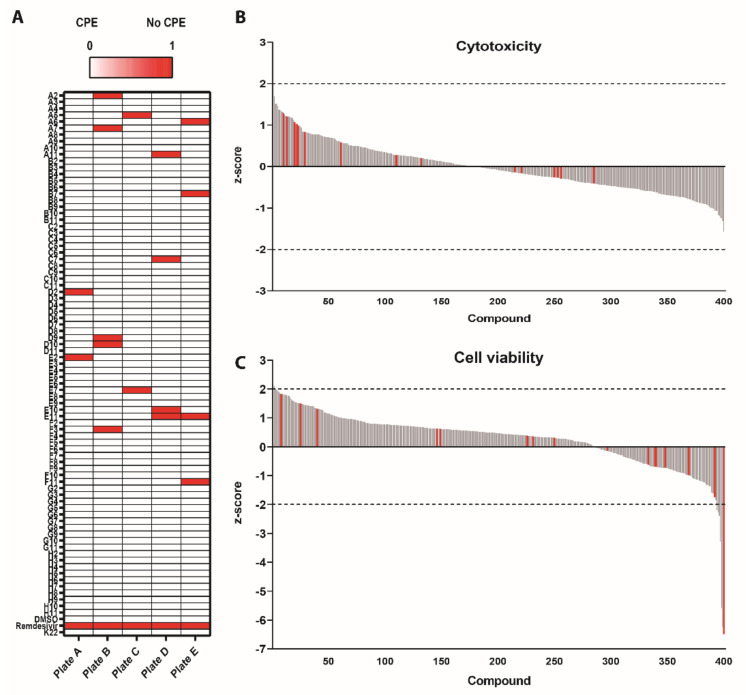
Cell survival upon infection with severe acute respiratory syndrome coronavirus 2 (SARS-CoV-2) (**A**), cytotoxicity (**B**) and viability (**C**) after incubation with pandemic response box compounds. The survival, cytotoxicity and viability were measured after 48 h of incubation on Vero-E6 cells at 37 °C in a humidified incubator with 5% CO_2_. The heatmap shows the position on the plates where survival was observed with a multiplicity of infection (MOI) of 0.01, which was scored from 0 to 1 (0 = cytopathogenic effects (CPE) and 1 = no CPE) (**A**). Calculated z-scores of each individual compound for cell cytotoxicity (**B**) and cell viability (**C**). A confidence interval of 95% (z-score of −2 or +2) was used as cutoff values, which are indicated with gray dashed lines. Each bar represents an individual compound sorted from high to low z-scores (*x*-axis). The red bars indicate the compounds that showed inhibition of SARS-CoV-2 during the survival screen. Results are shown as mean of two individual experiments performed in two technical replicates.

To identify potential antiviral compounds against SARS-CoV-2 replication, 201 antibacterial, 46 antifungal and 153 antiviral molecules included in the PRB were screened using a conservative concentration of 1 µM. Based on the documented inhibition of coronavirus replication, remdesivir was included as a positive control [8]. The compound K22 was also included in the analysis since it has been shown to inhibit a broad range of viruses including coronaviruses, arteriviruses, toroviruses as well as different members of the family Flaviviridae [17,24,25]. Vero E6 cells were pretreated for 2 h and subsequently infected with SARS-CoV-2 (MOI of 0.01) for 48 h in drug-containing medium. Cell survival was scored from 0 (virus-induced cytopathogenic effects; CPE) to 1 (no CPE), upon evaluation of SARS-CoV-2-induced CPE using crystal violet staining (Figure 1A). The cytotoxicity and cell viability were assessed on uninfected plates processed in parallel, to exclude detrimental effects of the compounds on the cells.

This survival screen resulted in a total of seventeen compounds that inhibited SARS-CoV-2-induced CPE (red). In parallel, cell viability and cell cytotoxicity assays were performed to exclude detrimental effects of each compound on the cells. The mean raw values were standardized by calculating the individual cell cytotoxicity and viability z-scores. Compounds exceeding a 95% confidence interval (z-score of −2 or +2) were regarded as nonviable hits. This showed that only a single compound caused significant cytotoxicity (Plate D, G11; z-score 3.12) (Figure 1B). In contrast, 10 compounds exceeded the cell viability cutoff, including the previously identified compound in Plate B, D10 (z-score: −6.498) (Figure 1C). Of note, this compound was also observed as a positive hit in the survival screen, but due to its impaired cell viability, it was excluded from further analysis. The sixteen remaining hit compounds are categorized as antifungal (three), antibacterial (six) and antiviral (seven) compounds (Appendix A), which, similarly to their vehicle control (DMSO) and remdesivir, did not influence cell cytotoxicity and cell viability. Interestingly, K22 did not show any inhibitory activity against SARS-CoV-2 at a concentration of 1 µM. These results highlight the relevance of a conservative and rapid screening of libraries containing compounds that could potentially inhibit SARS-CoV-2.

### 3.2. Antiviral Efficacy against SARS-CoV-2

**Figure 2:**
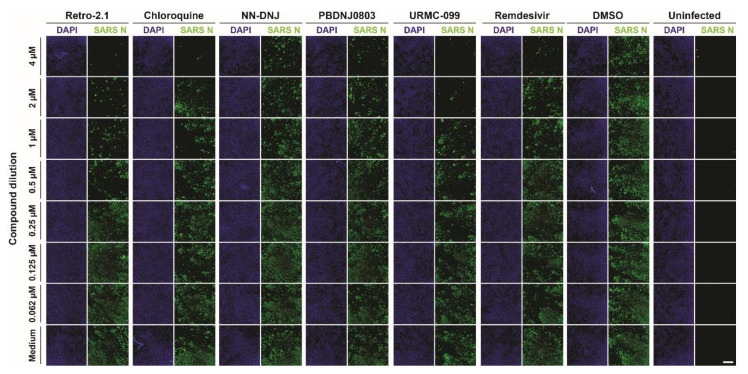
Assessment of the antiviral efficiency by immunofluorescence. To determine the efficiency of inhibition of each specific compound from the survival screen, a two-fold dilution series ranging from 4 µM to 0.062 µM was prepared following infection with SARS-CoV-2. Vero E6 cells were pretreated with the compound 2 h prior to infection with SARS-CoV-2 (MOI of 0.01) at 37 °C in a humidified incubator with 5% CO_2_. Cells were fixed 24 h post-infection, followed by immunostaining with the cross-reactive SARS-CoV nucleoprotein antigen (SARS N) and 4′,6-diamidino-2-phenylindole (DAPI). Remdesivir was included as a positive control. The images are representative of the results of three individual experiments. Scale bar: 1000 μm.

To further confirm and evaluate the extent of antiviral activity of the previously highlighted sixteen compounds, cells were pretreated with the selected compounds at concentrations ranging from 4 µM to 0.062 µM and infected with SARS-CoV-2 (MOI of 0.01). After 24 of infection, cells were fixed and processed for immunofluorescence analysis using the anti-SARS-CoV nucleocapsid protein antibody and DAPI (Figure 2).

**Figure 3:**
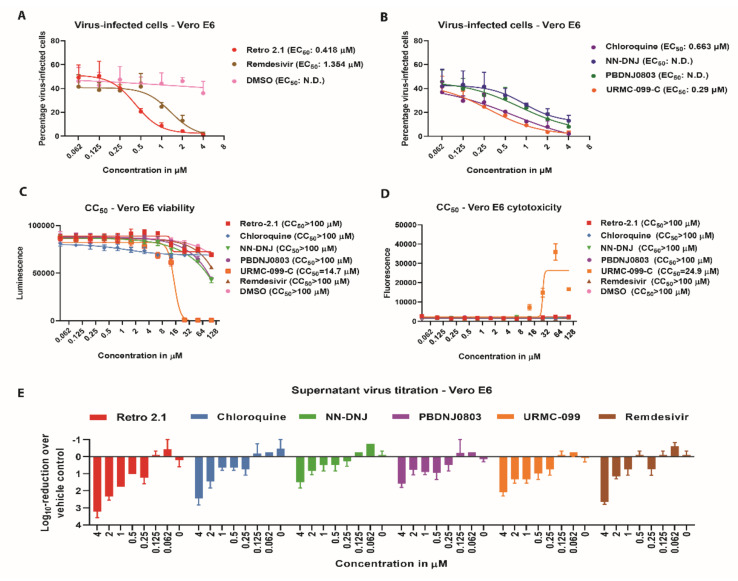
Half maximal effective concentration (EC_50_) determination of the five hit compounds showing inhibition against SARS-CoV-2. Vero E6 cells were pre-treated for 2 h with the indicated compound concentrations prior to SARS-CoV-2 infection (MOI 0.01) at 37 °C in a humidified incubator with 5% CO_2_. Following infection, cells were fixed and processed for immunofluorescence analysis. To determine the reduction in the percentage of virus-infected cells, the number of cells with a green fluorescent protein (GFP)-positive cytoplasmic signal (infected cells) was divided by the total number of cells (DAPI, nuclei). The percentage of virus-infected cells of Retro-2.1 was compared to remdesivir, DMSO (**A**) and chloroquine, *n*-nonyldeoxynojirimycin (NN-DNJ), PBDNJ0803 and URMC-099 (**B**). Abbreviations: N.D.: not determined. Results are displayed as means and SD of three independent experiments. The results of the half-maximum cytotoxicity concentration (CC_50_) based on viability (**C**) or cytotoxicity (**D**) are shown as means and SD from two individual experiments performed in two technical replicates. Corresponding supernatants were titrated by tissue culture infectious dose 50 (TCID_50_) to quantify the log_10_ reduction in SARS-CoV-2 titer (**E**). Results are displayed as means and SD of two independent experiments.

The efficiency of the selected compounds to inhibit SARS-CoV-2 (Figure 3A,B), as well as their individual effects on cell viability (Figure 3C), cytotoxicity (Figure 3D), and viral titer were assessed (Figure 3E). The EC_50_ values for each compound were inferred by calculating the percentage of virus-infected cells. This indicated that five out of sixteen candidate compounds, *n*-nonyldeoxynojirimycin (NN-DNJ), PDNJ0803, chloroquine, Retro-2.1 and URMC-099-C, inhibited SARS-CoV-2 in a dose-dependent manner (Figure 3C,D). Moreover, these five compounds showed EC_50_ values ranging from 0.29 µM to 0.63 µM, without increased cytotoxicity or decreased cell viability (Figure 3A–D). These results were corroborated by the dose-dependent reduction in viral titer, where Retro-2.1 displayed the strongest reduction (Figure 3E). Processing the samples at 48 h post-infection rather than 24 h post-infection showed that the inhibitory effect of all compounds is reduced at a later phase during infection (Figure S1). The remaining eleven compounds showed little or no inhibition at lower concentrations and were therefore excluded from further analysis (Figure S2).

In parallel to the inhibition efficiency, the half-maximum cytotoxicity concentration (CC_50_) was determined for each compound at concentrations ranging from 0.04 µM to 100 µM on Vero E6 cells. This demonstrated that the previously tested compounds were all well tolerated at concentrations up to 100-fold higher than the one used in the initial screen (1 μM) (Figure 3C,D, Appendix B). In contrast, only URMC-099-C displayed moderate cell cytotoxicity, resulting in a CC_50_ of 14.7 µM based on viability and 24.9 µM based on cytotoxicity, while all other compounds had a CC_50_ above 100 µM (Figure 3C,D, Appendix B). The resulting selectivity indexes (SI, the CC_50_-score divided by the EC_50_-score) showed that Retro-2.1 (SI = 239.2) is the most efficient and least cytotoxic inhibitor, followed by chloroquine (SI = 150.8) and URMC-099-C (SI = 50.7). The positive control remdesivir showed a SI of 73.8, while no SI values were calculated for NN-DNJ and PBDNJ0803 as the EC_50_-value could not be determined (Figure 3B, Appendix B). Combined, these results demonstrate the identification of five compounds of the pandemic response box library that inhibited SARS-CoV-2 in Vero E6 cells.

### 3.3. Inhibition Analysis of SARS-CoV-2 Infection on Calu-3 Cells

**Figure 4:**
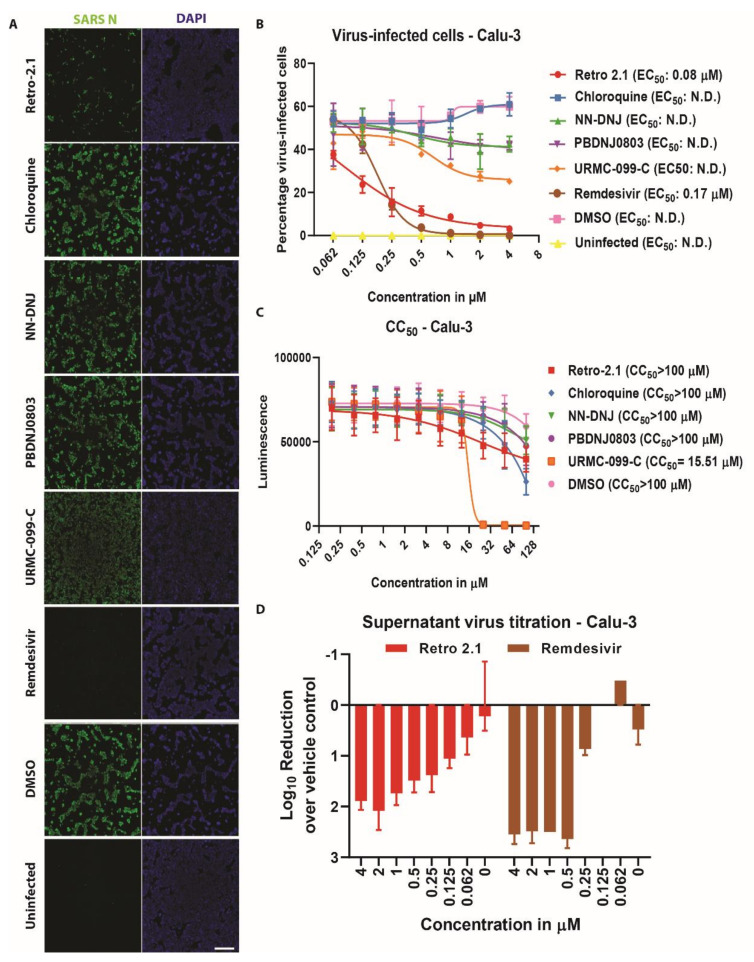
Analysis of the antiviral efficiency on Calu-3 cells. Calu-3 cells were pre-treated 2 h at the indicated concentration prior to SARS-CoV-2 infection (MOI 0.02) at 37 °C in a humidified incubator with 5% CO_2_. Cells were fixed 48 h post-infection, followed by immunostaining with the cross-reactive SARS-CoV nucleoprotein antibody (SARS N) and DAPI (**A**). The images are representative of the results of three individual experiments (only treatments with 4 μM are shown). Scale bar: 1000 μm. The half maximum inhibitory concentration (EC_50_) was determined using the percentage of infected cells and half maximum cytotoxicity concentration (CC_50_) was determined using cell viability (**B**, **C**). Results are displayed as means and SD of three individual experiments. Abbreviation: N.D.: not determined. Corresponding log_10_-reduction in SARS-CoV-2 viral titer for Retro 2.1 and remdesivir treatments (**D**).

To exclude potential cell-specific biases, the previous experiments were additionally performed using a human lung adenocarcinoma cell line (Calu-3), which recapitulates important biological aspects of the natural site of infection [26,27]. Interestingly, inhibition of SARS-CoV-2 replication was only observed upon treatment with Retro-2.1 (EC_50_: 0.08 μM) or with remdesivir (EC_50_: 0.17 μM), based on the percentage of virus-infected cells (Figure 4A,B). The other four compounds showed no viral inhibition in Calu-3 cells. Determination of the CC_50_ showed that most compounds were well tolerated by the Calu-3 cells with values >100 μM, except for URMC-099-C (CC_50_ = 15.51 μM) (Figure 4C). Consistently, the percentage of SARS-CoV-2-infected Calu-3 cells treated with Retro-2.1 (SI: 1250) or remdesivir (SI: 588) correlated with viral titers (Figure 4D). This highlights that from the five antiviral compounds identified using Vero E6 cells, only Retro-2.1 showed efficient inhibition of SARS-CoV-2 replication in both Vero E6 and Calu-3 cells.

### 3.4. Time-of-Addition

**Figure 5:**
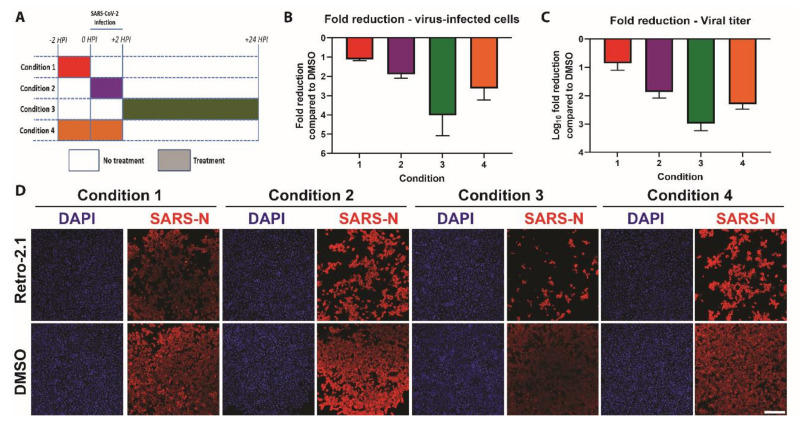
Time-of-addition of Retro-2.1. Time-of-addition was performed by Retro-2.1 treatments pre-, during and/or post-infection (**A**). Confluent Vero E6 cells were fixed 24 h post-infection (hpi) followed by processing for immunostaining with the cross-reactive SARS-CoV nucleoprotein antibody (SARS-N) and DAPI. The percentage of SARS-CoV-2-infected cells (**B**) and viral titers in the supernatant (**C**) were determined at 24 h post-infection. Graphs show means and SD from three individual experiments, performed in two technical replicates. (**D**) Representative images of the indicated conditions. Scale bar: 300 μm.

To determine which step of the viral replication cycle is affected by Retro-2.1, we performed a time-of-addition experiment. Confluent Vero E6 cells were either pre-treated with 10 μM of Retro-2.1, or an equal volume of vehicle control (DMSO), either two hours prior to infection, during infection, or two hours after infection. Alternatively, Retro-2.1 treatment was performed during both the pretreatment and virus infection steps (Figure 5A). At 24 h post-infection, the supernatant from each condition was titrated to determine the infectious viral titer, and the cells were processed for immunofluorescence analysis as described earlier.

The most pronounced inhibitory effect was observed when cells were treated with Retro-2.1 after SARS-CoV-2 infection (condition 3) (Figure 5B,D). In contrast, when cells were treated with Retro-2.1 prior to infection (condition 1), only a marginal reduction was observed. Treatment of cells during infection (condition 2) showed a more pronounced inhibition, which was comparable to condition 4, which combines treatment with Retro-2.1 prior and during infection. These results were corroborated by the viral titers analyzed at 24 h post-infection, demonstrating a >1000-fold reduction in viral titer in condition 3 (Figure 5C). These data indicate that Retro-2.1 likely interferes with the viral replication cycle of SARS-CoV-2 during a post-entry step.

## 4. Discussion

In this study, we have demonstrated that a conservative in vitro screening of 400 compounds from the PRB using two different cell lines refines the identification of effective antiviral candidate drugs targeting SARS-CoV-2. The first stage of the screening using Vero E6 cells led to the identification of five compounds displaying effective antiviral activity against SARS-CoV-2. This included the anti-malaria drug chloroquine and antibacterial Retro-2.1, as well as the antiviral compounds NN-DNJ, PDNJ0803, and URMC-099-C. These compounds showed a dose-dependent inhibition of SARS-CoV-2 infection. However, validation of these selected hits on the human lung cell line Calu-3 revealed that only Retro-2.1 efficiently inhibited SARS-CoV-2 replication. Time-of-addition analysis showed that Retro-2.1 likely impairs SARS-CoV-2 replication during a post-entry step of the viral replication cycle. Combined, these results demonstrate that robust evaluation of novel antiviral candidate drugs should be performed in different cell lines to exclude potential cell-dependent artefacts. K22, which exerts a well-documented inhibition of several coronaviruses including SARS-CoV and MERS-CoV, was, to our knowledge, not tested against SARS-CoV-2 [17,24,25]. Here, we show that K22 did not inhibit SARS-CoV-2 infection in Vero E6 cells, further emphasizing the requirements of rigorous screening conditions and robust controls during drug library screening assays.

In the light of the current pandemic, it has become evident that drug repurposing is a promising strategy that could rapidly help to mitigate SARS-CoV-2 infections. In our study, we employed a stringent survival screening approach using a library of 400 preselected compounds at a concentration of 1 μM. Other recent studies have employed different types of libraries and screening conditions, such as incubation time, cell density and MOI, but alike our study, most employed Vero E6 cells as an initial screening platform and used remdesivir as a positive control [28,29]. In line with these studies, we also identified chloroquine, which is approved for clinical use in the context of malaria treatment, as a potent antiviral compound against SARS-CoV-2, showing the reproducibility of these screens. Although the evaluation of chloroquine treatments against SARS-CoV-2 infections in clinical settings has been rapidly undertaken, it has been demonstrated that its antiviral activity on viral entry is restricted to Vero E6 cells [26]. Concordantly, in our study, we also observed that the impairment of SARS-CoV-2 replication by chloroquine was restricted to Vero E6 cells. Additionally, limited inhibition of SARS-CoV-2 by chloroquine was more recently confirmed by experimental infections of Syrian golden hamsters and rhesus macaques [30]. Altogether, these results emphasize the importance of the rigorous testing of antiviral compounds in multiple cell lines to exclude potential cell-based artefacts prior to initiating clinical trials.

In this study, we identified Retro-2.1 as a novel potent compound that inhibited SARS-CoV-2 replication in both Vero E6 and the human lung Calu-3 cell lines. Retro-2.1 has been previously shown to inhibit a broad range of viruses including enteroviruses, filoviruses, herpes simplex virus, vaccinia virus and polyomaviruses [31,32,33,34,35,36]. It has been suggested that Retro-2.1 remodels the intracellular distribution of syntaxins, which consequently alters vesicular retrograde transport between endosomes and the Golgi apparatus [37,38]. Moreover, convergent data have reported the strong dependence on cellular endomembranes and cellular trafficking pathways for efficient coronavirus replication. Several syntaxins (stxbp1, stxbp3, stx5a, and stx18) were identified in close proximity of the coronavirus replication and transcription complex during infection, some of which were further suggested to assist viral replication [39]. Consistent with these studies, our time-of-addition analysis revealed that Retro-2.1 substantially impairs SARS-CoV-2 infection during a post-entry stage of the viral replication cycle. Further investigations are required to define the precise molecular targets as well as the mode of action of Retro-2.1.

In summary, the stringent screening of 400 compounds identified Retro-2.1 as a potent antiviral compound against SARS-CoV-2 in vitro in different cell lines. This vindicates further efficacy and safety testing in other biologically relevant pre-clinical in vitro and in vivo models as novel intervention strategies against SARS-CoV-2 and other coronaviruses.

## Supplementary Materials

The following are available online at https://www.mdpi.com/2076-2607/8/12/1872/s1. Figure S1: The EC_50_ determination of the five top compounds after 48 h of incubation. Figure S2: EC_50_ determination of eleven compound hits that showed partial inhibition against SARS-CoV-2.

## Appendix A

**Table A1:** The sixteen compounds that showed inhibition of SARS-CoV-2 after the survival screen. Remdesivir was included as a reference drug control.

Plate Position:	Section	MVV-Number	CHEMBL ID	Reference	Name	Clinical Stage According CHEMBL	Chemical Formula
Plate A, D2	Antifungals	MMV1634386	CHEMBL3311228	https://www.ebi.ac.uk/chembl/compound_report_card/CHEMBL3311228/	Oteseconazole	3 (Phase III)	C23 H16 F7 N5 O2
Plate A, E2	Antifungals	MMV637528	CHEMBL64391	https://www.ebi.ac.uk/chembl/compound_report_card/CHEMBL64391/	Itraconazole	4 (approved)	C35 H38 Cl2 N8 O4
Plate B, A2	Antibacterials	MMV1483032	CHEMBL243644	https://www.ebi.ac.uk/chembl/compound_report_card/CHEMBL243644/	AC1MTT7T	0 (research)	C22 H18 N4 O4
Plate B, A7	Antibacterials	MMV637945	CHEMBL403	https://www.ebi.ac.uk/chembl/compound_report_card/CHEMBL403/	Sulbactam	4 (approved)	C8 H11 N O5 S
Plate B, D9	Antibacterials	MMV1582492	CHEMBL3109593	https://www.ebi.ac.uk/chembl/compound_report_card/CHEMBL3109593/	Retro-2.1	0 (research)	C23 H18 F N3 O S2
Plate B, F3	Antibacterials	MMV1582487	CHEMBL198796	https://www.ebi.ac.uk/chembl/compound_report_card/CHEMBL198796/	Decylphosphinate	0 (research)	C13 H28 N O4 P
Plate C, A5	Antibacterials	MMV1578576	CHEMBL1568820	https://www.ebi.ac.uk/chembl/compound_report_card/CHEMBL1568820/	-	0 (research)	C15 H12 F N3 O
Plate C, E7	Antibacterials	MMV000008	CHEMBL76	https://www.ebi.ac.uk/chembl/compound_report_card/CHEMBL76/	Chloroquine	4 (approved)	C18 H26 Cl N3
Plate D, A11	Antivirals	MMV1593513	CHEMBL408500	https://www.ebi.ac.uk/chembl/compound_report_card/CHEMBL408500/	*N*-Nonyl Deoxynojirimycin (NN-DNJ)	0 (research)	C15 H31 N O4
Plate D, C7	Antivirals	MMV690621	-	Patent: WO2006118607A2	NA for racemic	-	C18 H16 Cl N3 O
Plate D, E10	Antivirals	MMV1634401	CHEMBL1652119	https://www.ebi.ac.uk/chembl/compound_report_card/CHEMBL1652119/	PBDNJ0803	0 (research)	C20 H33 N O5
Plate D, E11	Antivirals	MMV002780	CHEMBL402487	https://www.ebi.ac.uk/chembl/compound_report_card/CHEMBL402487/	Noscapine	0 (research)	C22 H23 N O7
Plate E, A6	Antivirals	MMV1580482	CHEMBL2436978	https://www.ebi.ac.uk/chembl/compound_report_card/CHEMBL2436978/ Patent: WO 2014085795 A1	URMC-099-C	0 (research)	C27 H27 N5
Plate E, B7	Antivirals	MMV1593544	CHEMBL3752642	https://www.ebi.ac.uk/chembl/compound_report_card/CHEMBL3752642/	-	0 (research)	C36 H43 N3 O5
Plate E, E11	Antifungals	MMV002350	CHEMBL561	https://www.ebi.ac.uk/chembl/compound_report_card/CHEMBL561/	Lomefloxacin	4 (approved)	C17H19F2N3O3
Plate E, F11	Antivirals	MMV1580483	CHEMBL3960662	https://www.ebi.ac.uk/chembl/compound_report_card/CHEMBL3960662/	AZD0156	0 (research)	C26 H31 N5 O3
-	Control	-	CHEMBL4065616	https://www.ebi.ac.uk/chembl/compound_report_card/CHEMBL4065616/	Remdesivir	2 (Phase II)	C27 H35 N6 O8 P

## Appendix B

**Table A2:** The EC_50,_ EC_90_ and CC_50_ values of the five compounds that showed inhibition of SARS-CoV-2 during the compound dilution series on VeroE6-cells, including references to the mode of action of these compounds against other viruses. Remdesivir is included as a reference drug control. Abbreviations: N.D.: not determined.

Compound	EC_50_ in μM	EC_90_ in μM	CC_50_ in μM	Selective Index (SI)	Class	Examples of Susceptible Viruses	Literature Reference	Mode of Action	Administration	Tested on Cells or Organism	Molecular Structure
Retro-2.1	0.418	1.03	100	239.2	Retrograde transport inhibitor	Enterovirus Herpes simplex virus Filoviruses Vaccina virus Polyomavirus	[31,32,33,34,35,36]	Modulate intracellular vesicle transport (Enterovirus 71), Blocks entry (Herpes Simplex Virus 2) Blocks entry that follows by glycoprotein proteolysis (Filoviruses) Localization towards the ER (Polyomavirus) endosome-to-Golgi apparatus trafficking (Vaccina virus) induce cellular STX5 displacement from the Golgi upon treatment, leading to an inhibition of retrograde transport (Cytomegalovirus)	Prophylactic Therapeutic	Vero HeLa 293S Mice	
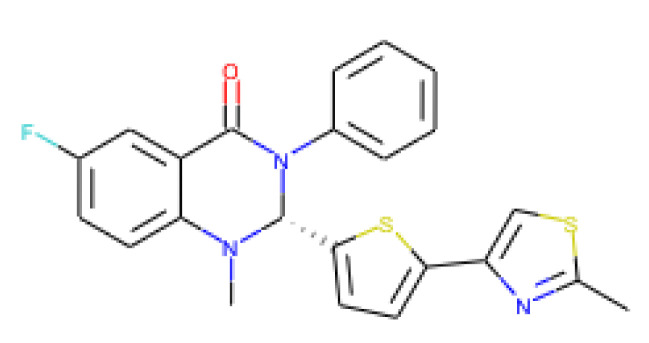
Chloroquine	0.663	10.44	100	150.82	Autophagic proteolysis inhibitor Endosomal acidification inhibitor	Herpes simplex virus Picornaviruses Poliovirus SARS-CoV HIV-1 Hepatitis A virus Hepatitis B virus Hepatitis C virus Influenza A virus Influenza B virus Bovine viral diarrhea virus 229E-CoV OC43-CoV Chikungunya virus Dengue virus Crimean-Congo Hemorrhagic fever Ebola virus Nipah virus Hendra virus Lassa virus Rabies virus	[40,41,42,43,44,45,46,47,48,49,50,51,52,53,54,55,56,57,58,59,60,61,62]	Raise endosomal pH (Picornavirus) Terminal glycosylation of the ACE2-receptor (SARS-CoV) Alter the glycosylation of the glycoprotein (HIV-1) Interference with endosomal acidifcation (Hepatitis C virus) Endosomal acidification (BVDV) P38 MAPK and ERK activation (229E)	Prophylactic Therapeutic	VeroE6 Vero 76 L132 MDBK Huh7 NS20 murine neuroblastoma cells B-SC-1 A549 Humans U937 MRC5 Guinea pigs	
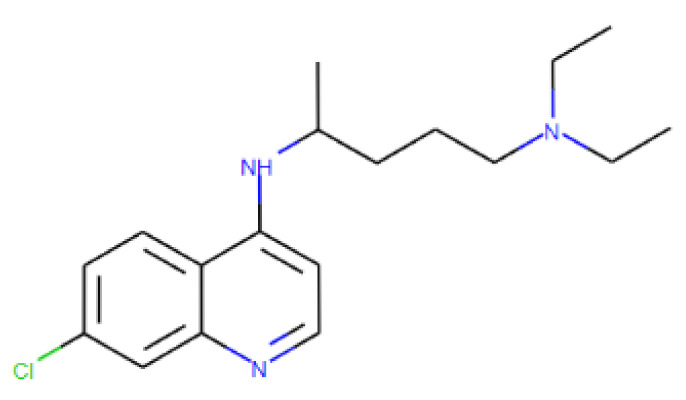
*N*-Nonyl Deoxyno Jirimycin (NNDNJ)	N.D.	N.D.	100	N.D.	ER α-glucosidase inhibitors	Bovine viral diarrhea virus West nile virus Dengue virus Hepatitis B virus Hepatitis C virus Flaviviruses Woodchuck hepatitis virus Ebola virus Tacaribe virus Junin virus SARS-CoV	[63,64,65,66,67,68,69,70,71] US patent: 9040488	Protein folding (Hepatitis B virus, Bovine viral diarrhea virus) Protein trafficking (Woodchuck hepatitis virus)	Prophylactic Therapeutic	MDBK BHK (-21) HepG2 Huh7.5 Woodchucks	
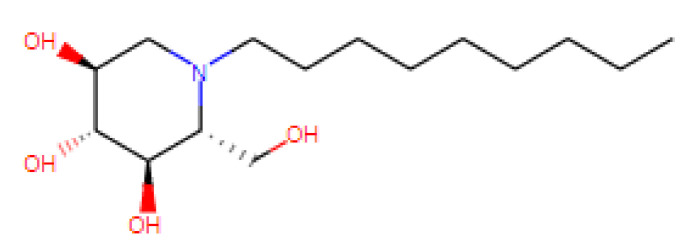
PBDNJ0803	N.D.	N.D.	100	N.D.	ER α-glucosidase inhibitors	Dengue virus Bovine viral diarrhea virus West nile virus Ebola virus Tacaribe virus Junin virus	[69,70] US Patent: 9040488	Protein folding (Bovine viral diarrhea virus)	Therapeutic	MDBK BHK Vero	
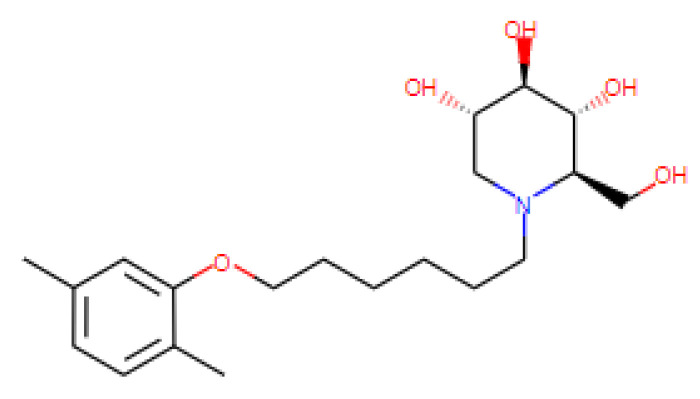
URMC-099-C	0.29	1.83	14.7	50.69	Mixed-lineage kinase 3 inhibitor	HIV-1 Zika virus	[72,73,74,75]	Anti-inflammatory Blocks phosphorylation of MLK3 Inhibits viral maturation in the endosome pro-viral effect in replication	Prophylactic Therapeutic	BV-2 microglial Mice Macrophages SNB-19	
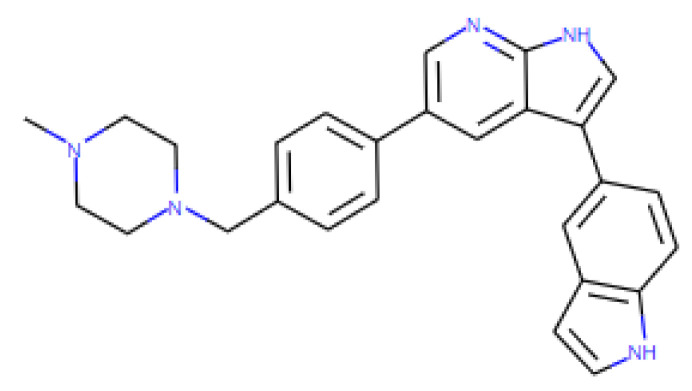
Remdesivir	1.354	3.39	100	73.85	Nucleoside analogue	Ebola virus Marburg virus SARS-CoV MERS-CoV Respiratory Syncitial Virus Junin virus Lassa virus Hendra virus Nipah virus Rift Valley Fever Virus Mumps virus Measles virus Coronaviruses Murine hepatitis virus	[18,76,77,78,79,80]	Interferes with the RNA dependent RNA polymerase activity	Prophylactic Therapeutic	Primary macrophages HeLa HFF-1 HMVEC-TERT Rhesus monkeys Huh7 Hep-2 U2OS Vero Mice Human airway epithelial	
