# Supplementary material for: Identification of an Antiviral Compound from the Pandemic Response Box that Efficiently Inhibits SARS-CoV-2 Infection In Vitro
Source: Microorganisms. 2020 Nov 26;8(12):1872. doi: 10.3390/microorganisms8121872 (PMC7760777; doi:10.3390/microorganisms8121872)
Supplement: Supplementary file 1 [file microorganisms-08-01872-s001.zip › Figure S1.docx]

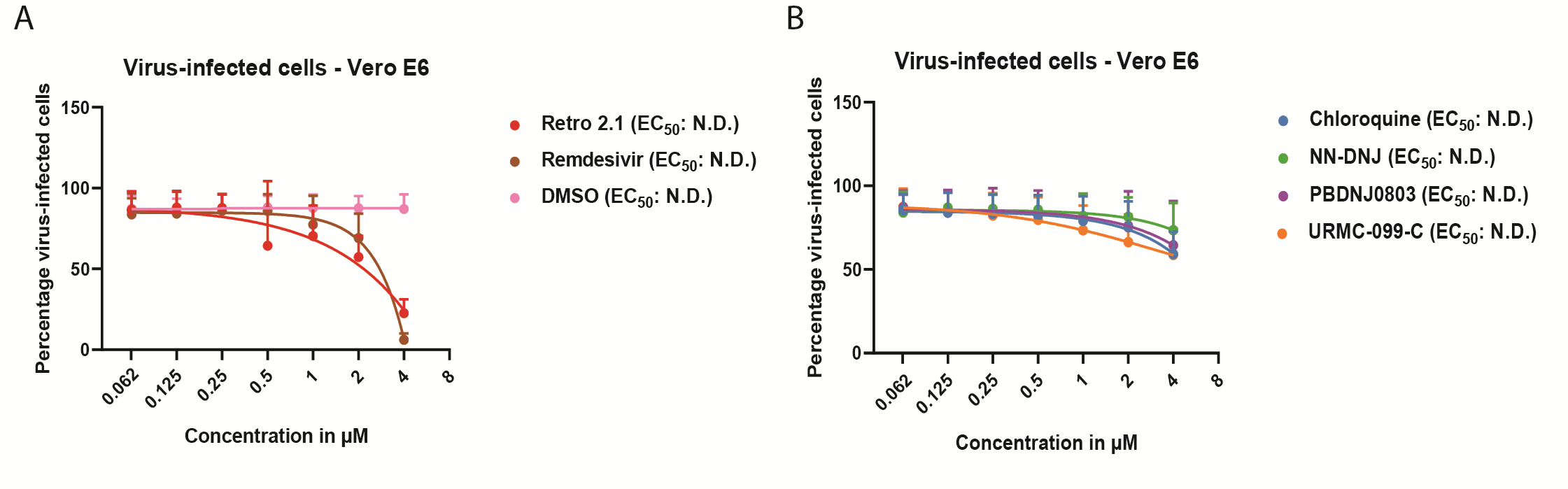


**Figure S1:**  Half maximal effective concentration (EC_50_) determination of the five hit compounds showing inhibition against SARS-CoV-2. Vero E6 cells were pre-treated for 2 h with the indicated compound concentrations prior to SARS-CoV-2 infection (MOI 0.01) at 37 °C in a humidified incubator with 5% CO_2_. Following infection, cells were fixed and processed at 48 h post-infection for immunofluorescence analysis. To determine the reduction in the percentage of virus-infected cells, the number of cells with a green fluorescent protein (GFP)-positive cytoplasmic signal (infected cells) was divided by the total number of cells (DAPI, nuclei). The percentage of virus-infected cells of Retro-2.1 was compared to remdesivir, DMSO (**A**) and chloroquine, *n*-nonyldeoxynojirimycin (NN-DNJ), PBDNJ0803 and URMC-099 (**B**). Abbreviations: N.D.: not determined. Results are displayed as means and SD of three independent experiments.
