# Supplementary material for: Identification of an Antiviral Compound from the Pandemic Response Box that Efficiently Inhibits SARS-CoV-2 Infection In Vitro
Source: Microorganisms. 2020 Nov 26;8(12):1872. doi: 10.3390/microorganisms8121872 (PMC7760777; doi:10.3390/microorganisms8121872)
Supplement: Supplementary file 1 [file microorganisms-08-01872-s001.zip › Figure S2.docx]

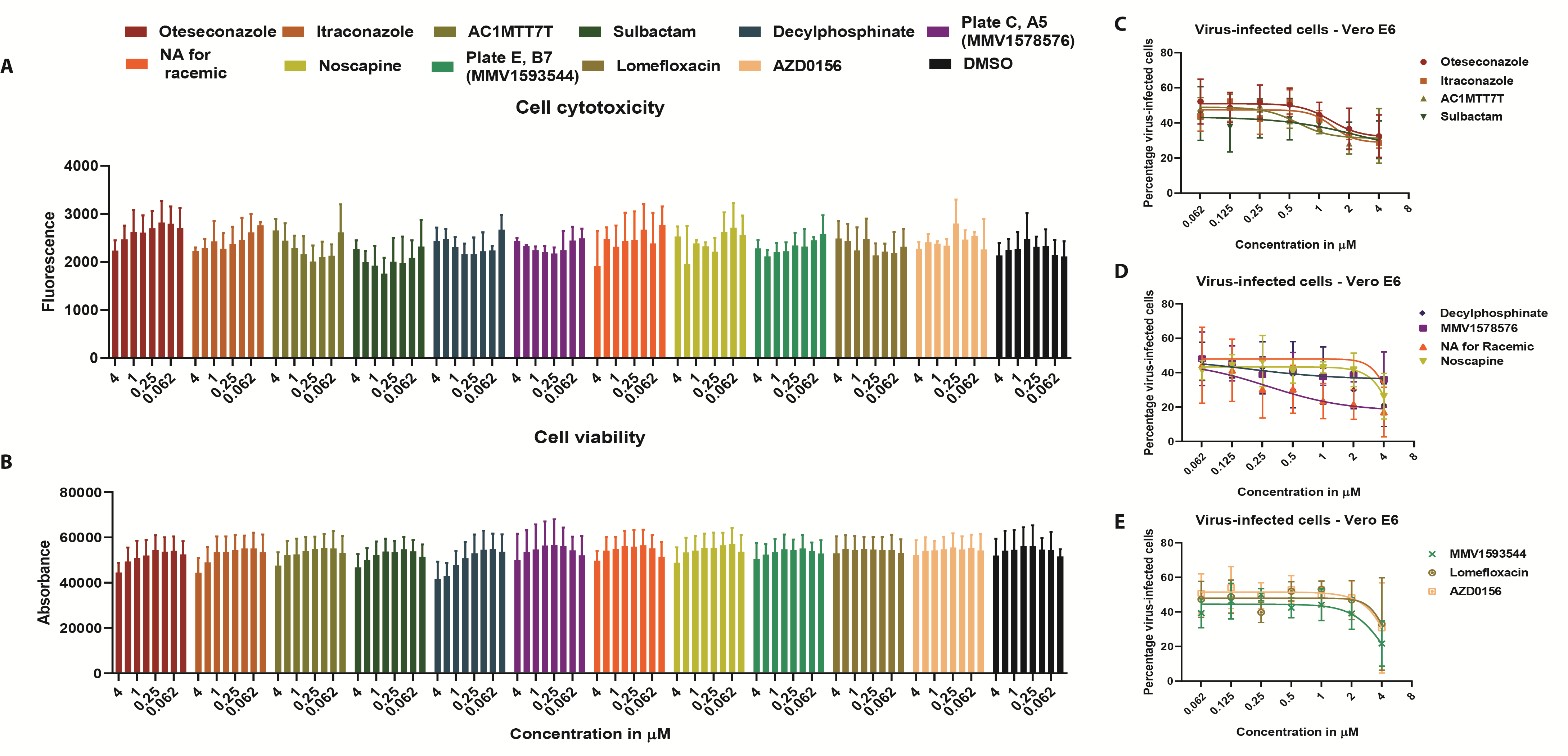


**Figure S2:** Half maximal effective concentration (EC_50_) determination of eleven compound hits that showed partial inhibition against SARS-CoV-2. The cell cytotoxicity **(A)** and cell viability **(B)** of the remaining 11 compound hits that showed partial inhibition during the compound dilution series were analyzed after 24 hours of incubation on Vero E6 cells at 37 °C in a humidified incubator with 5% CO2. Vero E6 cells were pre-treated for 2 h with the indicated compound concentrations prior to SARS-CoV-2 infection (MOI 0.01) at 37 °C in a humidified incubator with 5% CO2. Following infection, cells were fixed and processed for immunofluorescence analysis. To determine the reduction in the percentage of virus-infected cells, the number of cells with a green fluorescent protein (GFP)-positive cytoplasmic signal (infected cells) was divided by the total number of cells (DAPI, nuclei). The percentage of virus-infected cells of each compound dosage of Oteseconazole, Itraconazole, AC1MTT7T, Sulbactam **(C)**, Decylphosphinate, MMV1578576, NA for Racemic, Noscapine **(D)**, MMV1593544, Lomefloxacin and AZD0156 **(E)**. Results are displayed as means and SD of three individual experiments.
