# Supplementary figures and images for: Identification of an Antiviral Compound from the Pandemic Response Box that Efficiently Inhibits SARS-CoV-2 Infection In Vitro
Source: Microorganisms. 2020 Nov 26;8(12):1872. doi: 10.3390/microorganisms8121872 (PMC7760777; doi:10.3390/microorganisms8121872)

A

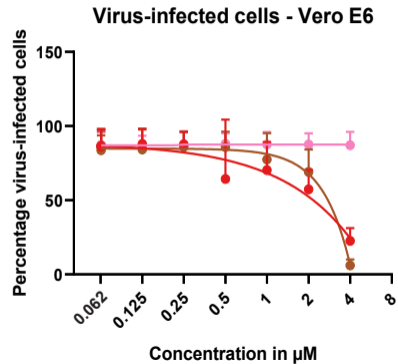

B

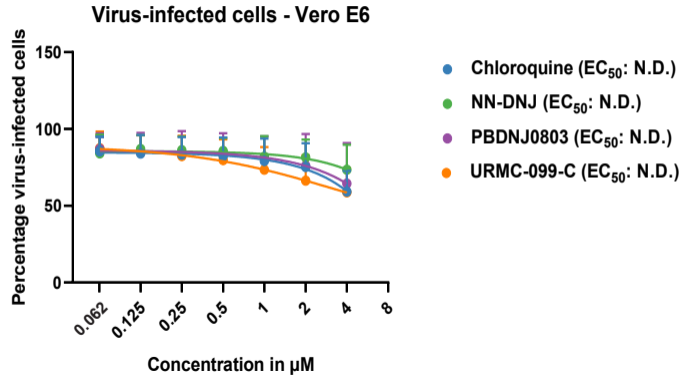

Supplement: Supplementary file 1 [file microorganisms-08-01872-s001.zip › S_Figure01.pdf]

**A**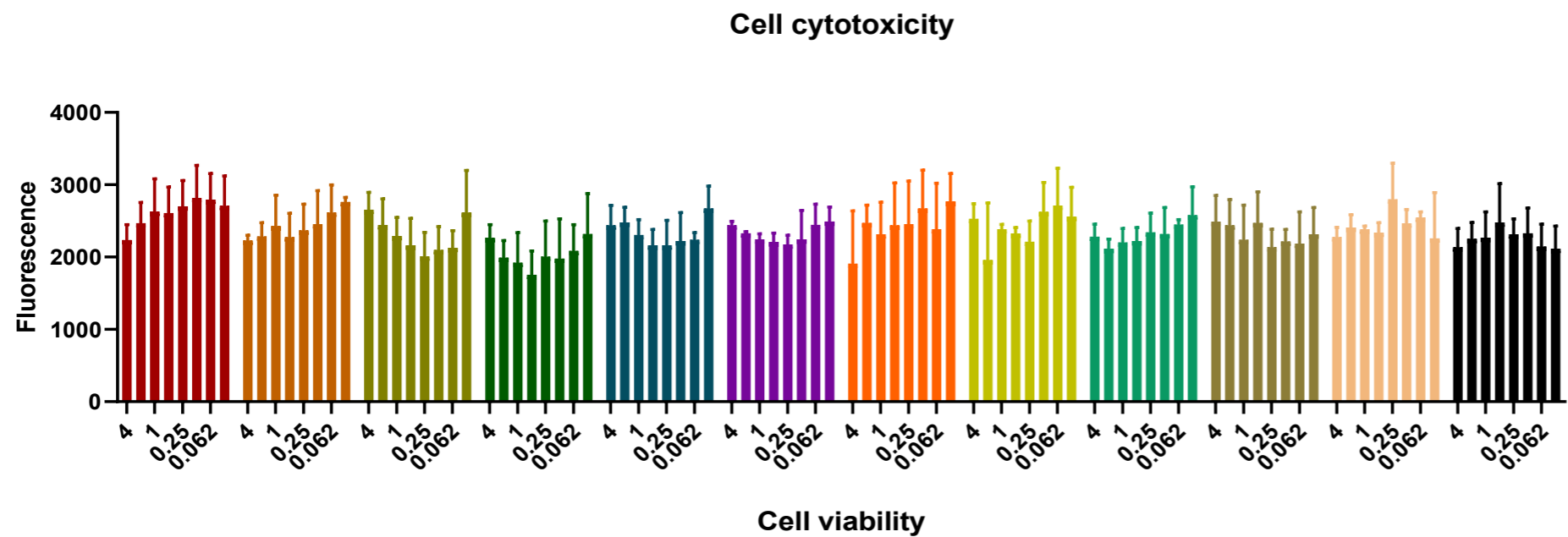**B**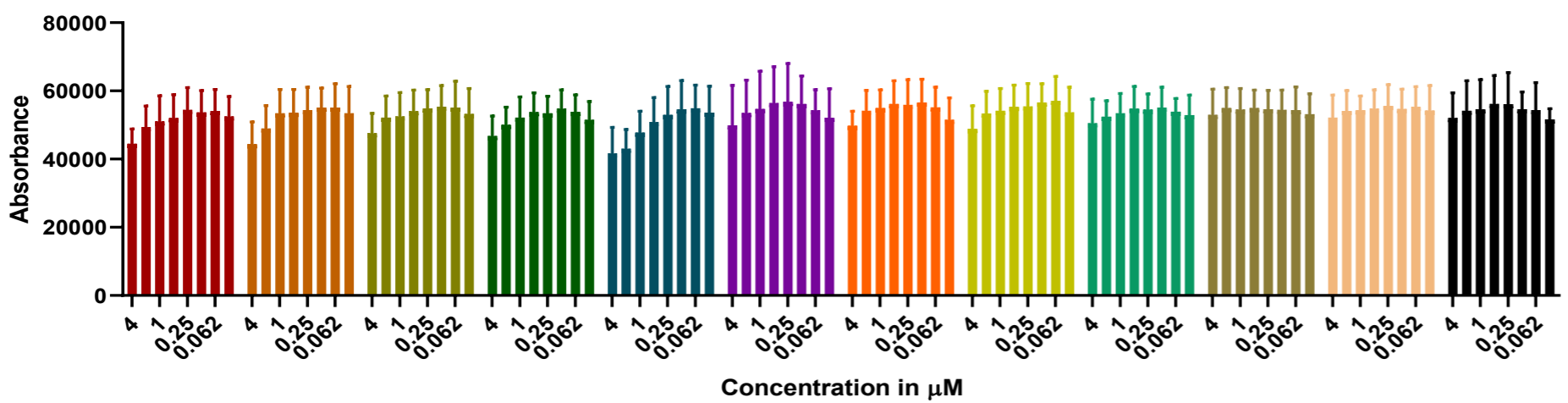**C**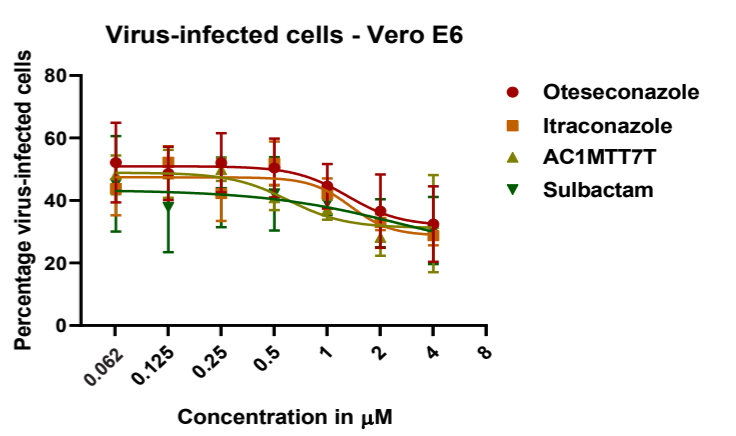**D**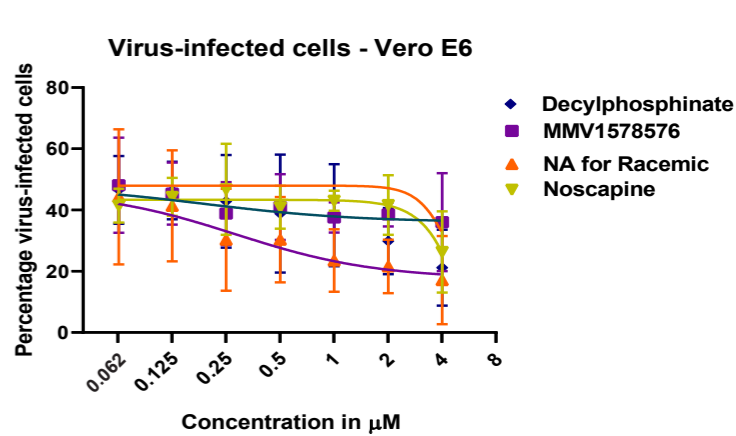**E**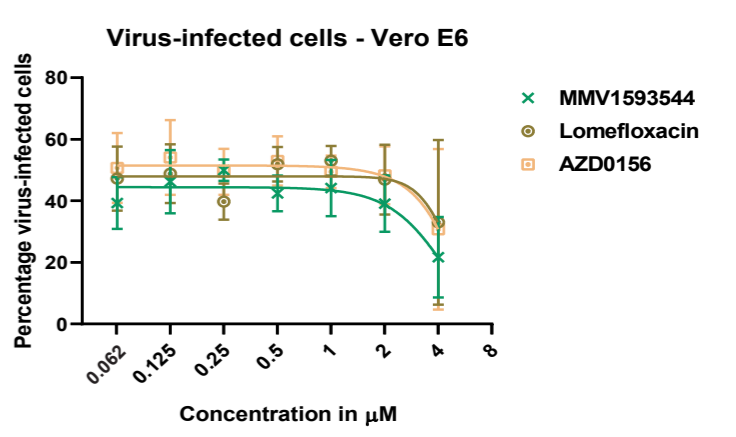

Supplement: Supplementary file 1 [file microorganisms-08-01872-s001.zip › S_Figure02.pdf]
